# Current Status of Gil-Vernet Trigonoplasty Technique

**DOI:** 10.1155/2008/536428

**Published:** 2008-07-29

**Authors:** Nasser Simforoosh, Mohammad H. Radfar

**Affiliations:** Urology & Nephrology Research Center, Shahid Labbafinejad Hospital, Shahid Beheshti University (M.C), Tehran 1666679951, Iran

## Abstract

Significant controversy exists regarding vesicoureteral reflux (VUR) management, due to lack of sufficient prospective studies. The rationale for surgical management is that VUR can cause recurrent episodes of pyelonephritis and long-term renal damage. Several surgical techniques have been introduced during the past decades. Open anti-reflux operations have high success rate, exceeding 95%, and long durability. The goal of this article is to review the Gil-Vernet trigonoplasty technique, which is a simple and highly successful technique but has not gained the attention it deserves. The mainstay of this technique is approximation of medial aspects of ureteral orifices to midline by one mattress suture. A unique advantage of Gil-Vernet trigonoplasty is its bilateral nature, which results in prevention from contralateral new reflux. Regarding not altering the normal course of the ureter in Gil-Vernet procedure, later catheterization of and retrograde access to the ureter can be performed normally. There is no report of ureterovesical junction obstruction following Gil-Vernet procedure. Gil-Vernet trigonoplasty can be performed without inserting a bladder catheter and drain on an outpatient setting. Several exclusive advantages of Gil-Vernet trigonoplasty make it necessary to reconsider the technique role in VUR management.

## 1. INTRODUCTION

Vesicoureteral reflux (VUR) is the most common urologic anomaly in children, affecting almost 1% of normal children [1, 2]. VUR is most commonly diagnosed during investigation of a child with history of urinary tract infection (UTI) [3, 4]. The frequency of VUR in children with UTI is 20–40% [5]. Evidence of renal involvement following UTI is more commonly found in children with VUR than children without VUR [6]. The combination of VUR and UTI predisposes children to acute pyelonephritis (APN) [7, 8]. Annual cost of hospitalization for pyelonephritis exceeds $180 000 000 in the U.S [9]. APN leads to subsequent renal scarring in 15–52% of the affected children [10, 11]. Renal scarring is an important risk factor for end stage renal disease (ESRD) and hypertension [2, 12]. ESRD is associated with reflux nephropathy in 3–25% of children and 10–15% of adults [5, 13]. 

Cooper and Austin have considered VUR as the “prostate cancer” of pediatric urology [14]. Significant controversy exists regarding VUR management, due to lack of sufficient prospective studies. The primary goal of VUR management is to prevent kidney damage. Management options include conservative medical treatment (antibiotic prophylaxis), and surgery (open or endoscopic). There are two important unanswered questions on who is a suitable candidate for antireflux surgery, either open or endoscopic, and which technique is the best for a patient. VUR resolves spontaneously with time in a large proportion of patients. Spontaneous resolution rate of VUR depends on reflux severity and patient's age at diagnosis, with higher rates at lower stages and younger ages. Reflux resolves in about 80%, 50%, and 30% of cases with VUR grades I to II, III, and IV, respectively [15–17]. The rationale for medical management is based on the potential of VUR for spontaneous resolution or decrease in severity, and on the ability of antibiotics to prevent UTIs and minimize renal damage until VUR ceases. Medical and surgical treatments of VUR have been compared in a meta-analysis, the results indicate that there is no significant difference in renal growth or scarring, and recurrence of UTI but the incidence of pyelonephritis is significantly reduced in surgical group [18]. The need for long-term daily medication, potential side effects, incompliance to the dosing regimen, and need for taking several voiding cystograms are disadvantages of medical management of VUR [19, 20]. The rationale for surgical management is that VUR can cause recurrent episodes of pyelonephritis and long-term renal damage. Despite controversies regarding indications of surgical treatment, expert opinion panels have described their recommendations on who is a good candidate for surgery. The AUA Pediatric Vesicoureteral Reflux Guidelines Panel recommended medical treatment as the initial management for all children with VUR diagnosed following UTI, with the exception of children over 1 year of age with grade V and older children with bilateral grade IV VUR. Indications for antireflux surgery include failure of renal growth, febrile UTI despite prophylaxis, noncompliance with medical management, the presence of new scars or deterioration of renal function, and reflux associated with congenital abnormalities of the ureterovesical junction [21]. Recommended indications are mostly based on expert opinions rather than on prospective controlled trials. To decide whether surgery is indicated for a particular child, the benefits and risks of surgical and medical management must be carefully assessed and individualized. In addition to the published indications for antireflux surgery, some other factors such as renal function, bladder function, and parental preference affect the final decision on selection of management options [22–24].

Antireflux surgical procedure may be performed endoscopically or open. The first report on antireflux surgery was published by Hutch in 1952 [25]. Several surgical techniques have been introduced during the past decades. Open antireflux operations have high success rate, exceeding 95%, and long durability. However, these techniques are invasive and impose a risk, although small, of surgical complications to the patient. Open techniques are categorized in two main groups; intravesical and extravesical. Politano and Leadbetter described an intravesical antireflux operation using ureteroneocystostomy in 1958 [26]. Other intravesical operations include ureteral advancement techniques; trigonal (Glenn-Anderson), (2) cross-trigonal (Cohen), and (3) medial advancement (Gil-Vernet). Extravesical ureteral reimplant was introduced by Lich and Gregoir in 1961 [27, 28].

**Figure 1:**
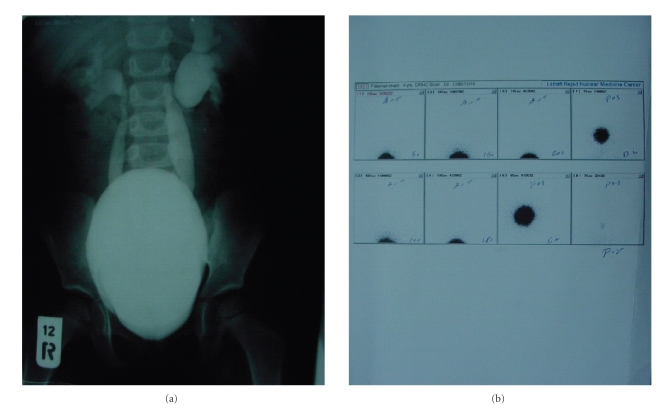
(a) Preoperative voiding cystoureterogram of a patient with bilateral high-grade vesicoureteral reflux. (b) Postoperative RNC of the patient reveals reflux resolution.

In the era of minimally invasive surgery, particularly for procedures with high success rate, capability of a technique to minimize surgery associated morbidities is significantly focused by most surgeons. The purpose of this article is to review the Gil-Vernet antireflux operation. Unfortunately, this simple and highly successful technique [29–31] has not gained the attention it deserves in urology field; it has not been evaluated by experts thoroughly. Since the technique was introduced by Gil-Vernet, the author and his colleagues have used this technique in more than one thousand pediatric and adult patients in their center, and published the results in several reports [32–34] (Figure 1). This article recalls the advantages of Gil-Vernet technique such as high success rate, being simple and rapid, and its potential to be performed on an outpatient setting.

## 2. GIL-VERNET ANTIREFLUX TECHNIQUE

**Figure 2:**
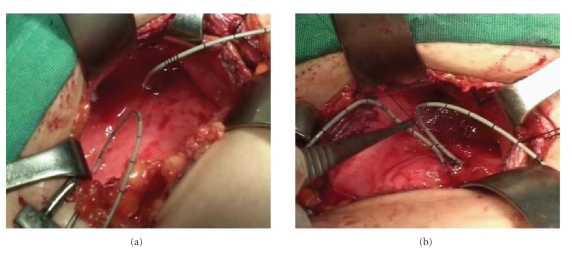
(a) Ureteral orifices of a patient with high-grade bilateral VUR located laterally (wide apart). (b) After performing Gil-Vernet trigonoplasty, ureteral orifices are located in the midline leading to effective detrusor support.

Gil-Vernet introduced his technique for antireflux surgery in 1984. He reported his experience in 38 patients with 94% success rate [35]. This technique is based on the sphincteric action of intrinsic muscular fibers of the transmural ureter, and additional muscular backing and intramural length provided by medial advancement of the ureters. Bladder mucosa is incised between ureteral orifices in a transverse fashion, and detrusor is taken down. Medial aspects of ureters are freed carefully from their surrounding tissues to be prepared and mobilized for advancement mattress sutures. Two 4-0 or 5-0 vycril mattress sutures, incorporating ureteral musculature, are placed on the medial aspect of the ureters. Mattress sutures bring ureters to the midline. It is highly influential to include ureteral musculature in the mattress sutures for prevention from late lateralization of ureters, technique failure, and VUR recurrence. Mucosa is closed vertically with interrupted chromic sutures, and the absorbable stitch is buried [35, 36] (Figure 2).

Ravasse et al. [37] reported their experience with Gil-Vernet technique in 30 children with primary vesicoureteral reflux in 1989. Patients were followed for 6–30 months. Reflux was corrected in all cases. Later several reports were published on the effectiveness of Gil-Vernet trigonoplasty. de Gennaro et al. [38] published their report on 51 children with 69 refluxing units. Mean patient age was 74 months (range from 4 months to 13 years). Reflux was grade II, III, and IV in 25, 39, and 25 refluxing units, respectively. Follow-up was performed for one year postoperatively. Surgery was successful in 97.7% of the patients. Reflux persisted in only one patient one year after the operation, in whom bilateral grade IV reflux was converted to unilateral grade III. In the study, patients were divided into 2 age groups: less than and greater than 3 years old. Success rate of surgery was 92.3% in children less than 3 years old and 100% in elder children. This finding is clearly in contrast to the assumption that Gil-Vernet technique is not appropriate for older children because of tenacious attachments of ureter in older ages [36].

Aghdas and Akhavizadegan [32] reported on applying Gil-Vernet technique in adult women with primary vesicoureteral reflux. A total of 39 women (mean age 29 years; range 18–65 years) with 49 refluxing units were included in the study. The Gil-vernet technique was successful in eliminating reflux in 48/49 renal units (97.95% success rate) and 38/39 patients (97.43% success rate). They concluded that Gil-Vernet antireflux surgery is highly successful in adult patients.

Zhao et al. [39] described Gil-Vernet's trigonoplasty in treating vesicoureteral reflux (VUR) in neurogenic bladders. They introduced a modification in technique as advancement of transmural ureters over the midline and crossing each other in the trigone. 43 refluxing units in 26 patients with neurogenic bladder underwent modified Gil-Vernet trigonoplasty. Refluxing units had grade I, II, III, IV, and V in 5, 7, 5, 18, and 8 patients, respectively. Reflux was unilateral in 9 patients, and bilateral in 17. Success rate of surgery was 95.3%, with a follow-up period of more than 2 years in most patients. The group concluded that modified Gil-Vernet's trigonoplasty might be a useful technique in the management of patients with VUR secondary to neurogenic bladder dysfunction.

The presence of a duplex ureter is one of the situations which complicate reflux [40]. Various antireflux techniques have been applied to correct reflux in duplex ureters. Kazemi-Rashed and Simforoosh [33] used Gil-Vernet technique to correct reflux in 12 patients with unilateral duplicated collecting system and 18 lower pole refluxing units. Reflux was bilateral in 50% of patients. Patient mean age was 5.6 years. Reflux was corrected or improved in 94% of units.

Garat et al. [41] reported an exclusive application of Gil-Vernet technique in exstrophy- epispadias patients. Reflux is associated with bladder exstrophy due to abnormal anatomic development of the distal ureter and to a pathologic bladder disposition. Mitchell's technique allows performing bladder closure, reconstruction of epispadias and the bladder neck in one single stage. However, pyelonephritis secondary to vesicouretral reflux is the most common postoperative complication. They applied Gil-Vernet as a first step of a bladder exstrophy repair followed by the Mitchell's technique. They concluded that combination of Gil-Vernet technique with the primary bladder closure could prevent the need for later surgical correction.

Several reports have been published on undertaking various antireflux techniques via a laparoscopic approach. Atala et al. [42] first described laparoscopic antireflux surgery using Lich-Gregoir technique in 4 mini pigs. Later, Ehrlich and Jantschek published the first reports on laparoscopic Lich-Gregoir surgery in human setting [43, 44]. Reports on laparoscopic cross-trigonal Cohen procedure have been published by Gill and Yeung [45, 46]. Okamura et al. reported their experience with endoscopic trigonoplasty but they could not achieve good results, because they did not exactly duplicate the principles used in open Gil-Vernet trigonoplasty [47]. Recently, we reported successful results following extraperitoneal laparoscopic trigonoplasty by complete duplication of Gil-Vernet open technique, achieving 93% success rate in all grades of reflux (II–IV) [34]. Regarding the simplicity of Gil-Vernet technique, it seems to be the most appropriate technique to be duplicated laparoscopically.

## 3. ADVANTAGES

### 3.1. Contralateral De novo reflux

Despite the high success rate of antireflux procedures to eliminate reflux in the operated ureter, secondary contralateral reflux is a relatively common complication occurring in 10–32% of cases [48]. Although de novo contralateral reflux resolves with time in most cases, 1.9–20% of children operated on for unilateral VUR have contralateral reflux after one year [49]. In one series, 13% of cases with contralateral reflux underwent surgical correction eventually [50]. Considerable attempts have been made to describe the possible mechanisms of developing contralateral reflux, but none of the proposed mechanisms are proven [48]. The risk for contralateral reflux is higher in patients with high grades of reflux, previous history of bilateral reflux, and duplex system [51, 52]. Some authors have recommended bilateral reimplantation for patients with the risk factors, but others have considered this as overtreatment [53]. One of the most important advantages of Gil-Vernet trigonoplasty is its bilateral nature. That is why in children with unilateral reflux; in contrast to other techniques, either open or endoscopic, Gil-Vernet trigonoplasty is the only technique that contralateral new reflux was not reported [54]. Furthermore, combination of Gil-Vernet with unilateral antireflux procedures has been recommended in several studies. Liard et al. [48] recommended contralateral meatal advancement based on the Gil-Vernet technique in patients undergoing Cohen antireflux procedure. Caione et al. [53] reported another series of patients, in whom contralateral meatal advancement was undertaken in combination with Cohen, Politano-Leadbetter, and Glenn-Anderson. Consequently, contralateral reflux was seen in none of the patients.

### 3.2. Ureteroscopy

A main advantage of Gil-Vernet procedure is that later catheterization of and retrograde access to the ureter can be performed normally [53]. In Cohen procedure, a highly popular and successful antireflux technique, the ureteral orifice is relocated. Alteration of the normal course of the ureter makes retrograde access to the ureter difficult [55]. Regarding almost all ureteral stones are currently treated endoscopically, the importance of easy endoscopic access cannot be overemphasized.

### 3.3. Catheter-free

Need for indwelling Foley catheter has been considered as a disadvantage of intravesical antireflux operations [13]. Since in extravesical Lich-Gregoir technique a catheter does not need to be left in bladder, it is associated with reduced bladder spasm and discomfort, and hematuria [13]. However, urinary retention occurs in 8%–35.6% of children after extravesical reimplantation [56, 57]. Recently, a study has described Gil-Vernet trigonoplasty without inserting a bladder catheter in 65 children with 103 refluxing units. VUR was corrected in 94.1% of patients, with no considerable complications. The authors concluded that Gil-Vernet surgery could be performed on an outpatient setting [58].

### 3.4. Obstruction

The most serious complication of antireflux procedure, which may require a reoperation, is ureterovesical junction obstruction (UVJO) [22]. Totally UVJO is seen in 2.5% of children underwent antireflux surgery, 2–4% after Lich-Gregoir technique, and 1% after Politano-Leadbetter [22, 59, 60]. In a report by Kliment et al. [61] on 60 children underwent Gil-Vernet surgery, UVJO was seen in none of the cases. To our knowledge, there is no report of UVJO following Gil-Vernet procedure. It is because the technique preserves the integrity of ureterovesical junction.

## 4. CONCLUSION

Among open surgical techniques commonly used, Gil-vernet trigonoplasty seems to be one of the least invasive. It is simple, safe, highly successful, with the advantage of possible ureteroscopy in the era of Endourology. Contralateral reflux will not follow this technique in managing unilateral reflux which is a unique advantage of this technique. The procedure could be applied in various particular situations such as neurogenic bladder, adult patients, duplex ureter, and exstrophy-epispadias. Simplicity of the technique allows undertaking the surgery laparoscopically. Several exclusive advantages of Gil-Vernet trigonoplasty make it necessary to reconsider the technique role in VUR management.
